# Ethnic differences in cardiometabolic risk profile in an overweight/obese paediatric cohort in the Netherlands: a cross-sectional study

**DOI:** 10.1186/1475-2840-8-2

**Published:** 2009-01-19

**Authors:** Mariska van Vliet, Inès A von Rosenstiel, Roger K Schindhelm, Desiderius PM Brandjes, Jos H Beijnen, Michaela Diamant

**Affiliations:** 1 Department of Paediatrics, Slotervaart Hospital, Amsterdam, the Netherlands; 2 Department of Endocrinology/Diabetes Centre, VU University Medical Centre, Amsterdam, the Netherlands; 3 Department of Clinical Chemistry, Isala Clinics, Zwolle, the Netherlands; 4 Department of Internal Medicine, Slotervaart Hospital, Amsterdam, the Netherlands; 5 Department of Pharmacy & Pharmacology, Slotervaart Hospital, Amsterdam, the Netherlands

## Abstract

**Background:**

Differences in prevalence of cardiometabolic risk factors between different ethnic groups are largely unknown. We determined the variation in cardiometabolic risk profile according to ethnicity in a cohort overweight/obese Dutch children.

**Methods:**

An oral glucose tolerance test was performed in 516 overweight/obese Dutch children of multi-ethnic origin, attending an obesity out-patient clinic of an urban general hospital (mean age 10.6 ± 3.2; 55.2% boys). Anthropometric parameters and blood samples were collected, and the prevalence of (components of) the metabolic syndrome (MetS) and insulin resistance were determined in each ethnic group.

**Results:**

Major ethnic groups were Dutch native (18.4%), Turkish (28.1%), and Moroccan (25.8%). The remaining group (27.7%) consisted of children with other ethnicities. Turkish children had the highest mean standardized BMI compared to Dutch native children (*P *< 0.05). As compared to Moroccan children, they had a higher prevalence of MetS (22.8% vs. 12.8%), low HDL-cholesterol (37.9% vs. 25.8%), hypertension (29.7% vs. 18.0%) and insulin resistance (54.9% vs. 37.4%, all *P *< 0.05). Although Turkish children also had higher prevalences of forementioned risk factors than Dutch native children, these differences were not statistically significant. Insulin resistance was associated with MetS in the Turkish and Moroccan subgroup (OR 6.6; 95%CI, 2.4–18.3 and OR 7.0; 95%CI, 2.1–23.1, respectively).

**Conclusion:**

In a Dutch cohort of overweight/obese children, Turkish children showed significantly higher prevalences of cardiometabolic risk factors relative to their peers of Moroccan descent. The prospective value of these findings needs to be established as this may warrant the need for differential ethnic-specific preventive measures.

## Background

Currently 10.6% of the Dutch population consists of non-Western immigrants, with people of Turkish (21.2%), Surinamese (19.2%) and Moroccan descent (19.0%) being the major minority groups [1]. The globally increasing prevalence of obesity and its accompanying health risks, such as type 2 diabetes mellitus and cardiovascular disease (CVD) [2-4], concerns all racial groups, including the aforementioned minority groups. Compared to the inhabitants of their country of origin, immigrant groups often have increased risk of developing CVD. For example, data on Japanese immigrants living in the United States indicate that a westernized lifestyle aggravates the risk factors for atherosclerosis and its progression [5]. Although a limited number of studies have directly examined the extent to which the prevalence of obesity and cardiometabolic risk factors vary by ethnicity, certain ethnic groups have been identified as having a greater susceptibility for CVD [6]. A recent study in the Netherlands found that female Turkish and Moroccan migrants had a two-to-three fold higher risk of being overweight [7], and similar differences have been reported in Turkish and Moroccan children in comparison with Dutch native children [8]. Apart from overweight, little is known about the prevalence of cardiometabolic risk factors in children among ethnic groups in the Netherlands. Studies on cardiometabolic risk factors which compared Dutch native and Non-Western migrant adults showed a lower prevalence of dyslipidaemia and a higher prevalence of diabetes and CVD among Turks, whereas the prevalence of CVD was lower in Moroccans, despite the higher prevalence of diabetes [7,9]. Ethnic differences may already be present in migrant children and could predict the risk of obesity and CVD in adulthood. Indeed, a number of studies have stressed the relevance of cardiometabolic risk factors at a young age for the development of CVD in later life [10-13].

A way to estimate cardiometabolic risk in both adults and children is to identify the presence of (components of) the 'metabolic syndrome' (MetS), a clustering of cardiometabolic disorders, including (central) obesity, impaired glucose metabolism, hypertension and dyslipidaemia [14-16]. In the various available definitions of MetS, adjustment for ethnicity is not applied, while on bases of previously found differences in prevalence of MetS among ethnic adult populations, adjustment may be required [13].

In the present study, we determined the prevalence of the metabolic syndrome and its components, according to a paediatric definition, in three major ethnic groups in a Dutch multi-ethnic cohort of obese and overweight children.

## Methods

### Study population and study protocol

In the period 2004–2008, data from a cohort children (aged 3–18 years), who were overweight or obese (Z-BMI ≥ 1.1) and who visited an urban general hospital in Amsterdam (Slotervaart Hospital), were collected according to a prevailing treatment protocol. Children with (suspected) syndromes and with type 1 diabetes or with secondary causes of obesity such as hypothyroidism, hypogonadism and pituitary disorders, or children who used glucose- or lipid-lowering medication, corticosteroids (chronically) or drugs acting on the central nervous system, were excluded from the study. All subjects had no history of alcohol abuse and serologic tests for hepatitis B or C virus were all negative. In total, 516 children of multi-ethnic origin, who were overweight or obese, were included in the study.

During the first visit, a detailed history and physical examination was performed, including blood pressure measurements, and assessment of height, weight, waist circumference, and pubertal stage according to Tanner [17]. Waist circumference was measured in accordance with a previously described method [10]. During the second visit, each child underwent an oral glucose tolerance test (OGTT; 1.75 g/kg with a maximum of 75 g). Blood samples for lipid levels, insulin, and glucose were taken before the OGTT (fasting levels) and two hours after glucose intake (glucose only).

### Definitions

Ethnicity was defined as Dutch if both parents were Dutch native, and Moroccan or Turkish in case both parents were from that specific country. Children with other ethnicities, and children with parents of different origins were collected in a separate group, hereafter referred to as the 'other' group. This group comprises children from areas all over the world i.e. Africa (Egypt, Somalia, Senegal, Ghana, Congo), The Caribbean (Dominican Republic, Suriname, Jamaica, Antilles), South America (Argentina, Chile), Middle East (Lebanon, Iraq, Iran), South Asia (India, Pakistan, Kurdistan, Sri Lanka), South East Asia (Philippines, Indonesia), China and Europe (Portugal, Bosnia). This brings the total number of ethnicities in this group to about twenty-three, which leaves too small numbers per ethnic group to analyse separately.

BMI was standardized using Z-scores (Z-BMI) according to Dutch reference values for Dutch native, Turkish and Moroccan children [18-20]. Waist circumference measurements were standardized according to Dutch reference values (Z-WC) [21]. Impaired glucose tolerance (IGT) was defined as a 2 h-glucose ≥ 7.8 and <11.0 mmol/L and impaired fasting glucose (IFG) was defined as a fasting glucose 5.6 to 6.9 mmol/L [13]. Lipid levels were evaluated with reference values adjusted for age and sex [22], with cut-off points above the 95^th ^percentile for total cholesterol, LDL-cholesterol and triglycerides, and below the 5^th ^percentile for HDL-cholesterol. Blood pressure values were considered abnormal when values were above the 95^th ^percentile, according to European reference values for height and sex [23].

MetS was diagnosed according to the definition proposed by Weiss *et al *on defining MetS in children and adolescents [13]. MetS was established when three or more of the following criteria were present: obesity (BMI above 97^th ^percentile for age and sex), a triglyceride level above the 95^th ^percentile [22], an HDL-cholesterol level below the 5^th ^percentile [22], a diastolic or systolic blood pressure above the 95^th ^percentile [23], and IGT. Insulin resistance was calculated according to the Homeostasis Model Assessment for Insulin Resistance (HOMA-IR): fasting plasma insulin (μU/L) × fasting glucose (mmol/L)/22.5 [24]. Insulin resistance was defined as HOMA-IR ≥ 3.5 [25]. Pancreatic β-cell function was expressed as HOMA-B, computed as (20 × insulin (μU/L))/(fasting glucose (mmol/L) – 3.5) [24].

### Laboratory analyses

Plasma glucose levels and fasting triglyceride, HDL-cholesterol and total cholesterol levels were measured by the SYNCHRON LX 20 (Beckman Coulter, USA). Low-density lipoprotein (LDL-) cholesterol was calculated with the Friedewald-formula [26]. Plasma insulin levels were measured by an immunoluminometric assay (Immulite 200 system, DPC, Los Angeles, USA; intra-assay variation: 3–6%, inter-assay variation: 3–5%).

### Statistical analyses

Mean (standard deviation), median (interquartile range) or percentages are shown. Results were stratified by ethnic group. Differences between group proportions were analyzed by χ^2 ^tests, and differences between groups were analyzed by analyses of variance (ANOVA) with post-hoc Bonferroni tests or by independent Student's t-tests. Variables with a skewed distribution were log transformed for the analyses. Logistic regression analysis was performed to assess the associations between insulin resistance and (components of) MetS. Results of these analyses are expressed as odds ratios (OR) and 95% confidence intervals (95%CI). To identify possible confounders in these analyses, we introduced sex, age, pubertal stage and Z-BMI stepwise. Aforementioned variables were considered confounders when causing a change in the regression coefficient beta of more than 10%.

A *P-*value < 0.05 was considered statistically significant. All analyses were performed with SPSS, version 15.0 (for Windows).

## Results

### Baseline characteristics

The cohort consisted of 516 children (55.2% boys, 41.5% prepubertal, mean age 10.6 ± 3.2 years), with the main ethnic groups being Dutch native (18.4%), Turkish (28.1%), Moroccan (25.8%), and a group consisting of at least 23 ethnicities all represented by relatively small numbers of children, designated as 'other' (27.7%). Due to the small numbers of the respective ethnic groups in the 'other' group, no separate analyses could be made, but to provide a complete overview the data of this mixed group is also included in the Tables and Graphs.

Table 1 shows the baseline characteristics of the three main ethnic groups. Dutch native children had the lowest mean Z-BMI, compared to Turkish children (2.6 ± 0.5 vs. 2.8 ± 0.5, *P *= 0.024). Turkish children and children in the 'other' group had highest fasting insulin levels, HOMA-IR, HOMA-B and triglycerides as compared to Moroccan children (all *P *< 0.05). In addition, Turkish children had lowest HDL-cholesterol levels (*P *= 0.007) and highest diastolic blood pressure (*P *= 0.015), compared to their Moroccan peers.

**Table 1:** Anthropometric and cardiometabolic characteristics according to ethnicity

	Dutch Native	Turkish	Moroccan	Other	*P*-value
N (%)	95 (18.4)	145 (28.1)	133 (25.8)	143 (27.7)	-

Male (%)	49.5	60.7	56.4	52.4	0.19

Pubertal (%)	55.8	63.4	57.1	56.6	0.80

Age (years)	11 ± 3	11 ± 3	10 ± 3	11 ± 3	0.23

Z-BMI	2.6 ± 0.5	2.8 ± 0.5*	2.8 ± 0.5	2.8 ± 4.5	0.03

Z-WC	3.8 ± 1.2	4.1 ± 1.1	3.9 ± 1.3	3.8 ± 1.0	0.16

SBP (mmHg)	116 ± 11	116 ± 12	113 ± 11	115 ± 12	0.11

DBP (mmHg)	71 ± 8	73 ± 9^†^	70 ± 8	71 ± 8	0.02

Fasting plasma glucose (mmol/L)	5.2 ± 0.4	5.3 ± 0.4	5.3 ± 0.4	5.3 ± 0.4	0.17

2-h plasma glucose (mmol/L)	5.8 ± 0.9	5.9 ± 1.0	5.7 ± 0.9	5.9 ± 1.0	0.083

Fasting plasma insulin (pmol/L)	95 (65–165)	112 (83–167)^‡^	90 (58–138)	112 (71–188)^†^	0.002

HOMA-IR	3.1 (2.0–5.4)	3.6 (2.6–5.7)^‡^	3.0 (1.8–4.7)	3.5 (2.4–6.1)^†^	0.003

HOMA-B	159 (109–258)	177 (120–251)^‡^	138 (99–204)	174 (112–288)^†^	0.004

Total cholesterol (mmol/L)	4.4 ± 0.9	4.3 ± 0.8	4.2 ± 0.8	4.4 ± 0.7	0.23

HDL-cholesterol (mmol/L)	1.1 (0.9–1.2)	1.0 (0.9–1.2)^‡^	1.1 (0.9–1.3)	1.0 (0.9–1.2)	0.014

LDL-cholesterol (mmol/L)	2.8 ± 0.6	2.8 ± 0.7	2.7 ± 0.7	2.9 ± 0.6	0.075

Triglycerides (mmol/L)	0.9 (0.6–1.3)	0.9 (0.6–1.3)^‡^	0.7 (0.5–1.0)	1.1 (0.7–1.2)^†^	0.004

Data are mean ± SD and n (%) or median (interquartile range) for variables with a skewed distribution. *P*-values are given for comparisons between ethnic groups, tested with ANOVA.

SD-standard deviation; Z-BMI-standard deviation score of BMI; Z-WC-standard deviation score of waist circumference; HOMA-IR-Homeostasis Model Assessment for Insulin Resistance; HOMA-B-Homeostatis Model Assessment for pancreatic β-cell function

*P < 0.05 compared to Dutch native children, ^†^*P < 0.05 compared to Moroccan children*, ^‡^*P *< 0.01 compared to Moroccan children.

The difference in mean LDL-cholesterol levels between Dutch native children and Moroccan children was borderline significant (*P *= 0.054).

### Prevalence of (components of) the metabolic syndrome

**Figure 1:**
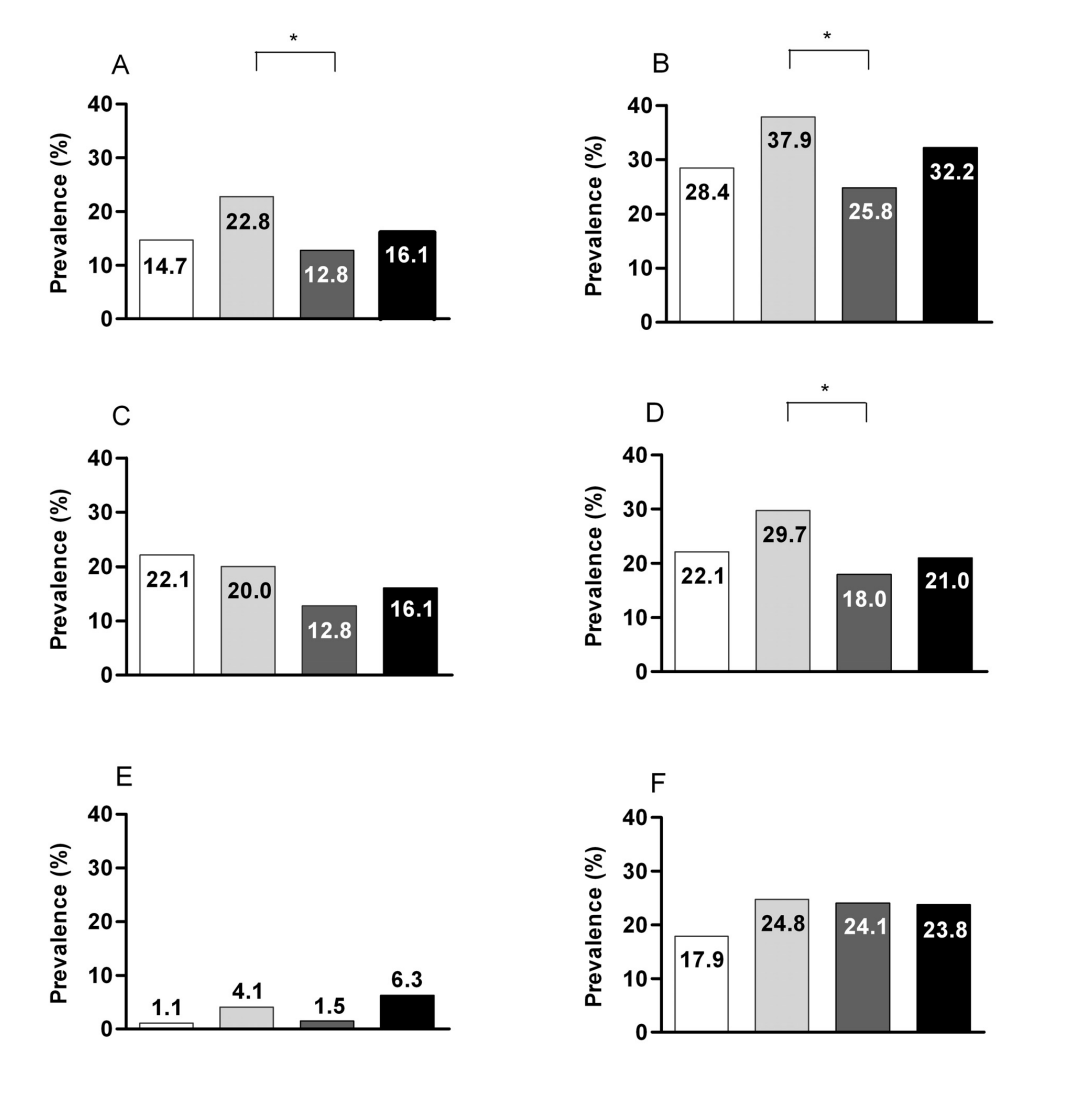
**Prevalence of the metabolic syndrome (A), low HDL-cholesterol (B), high triglycerides (C), hypertension (D), impaired glucose tolerance (E) and impaired fasting glucose (F) according to ethnicity; Dutch native (white bars), Turkish (light grey bars), Moroccan (dark grey bars), and 'other' (black bars)**. * χ^2 ^test, all *P *< 0.05.

**Figure 2:**
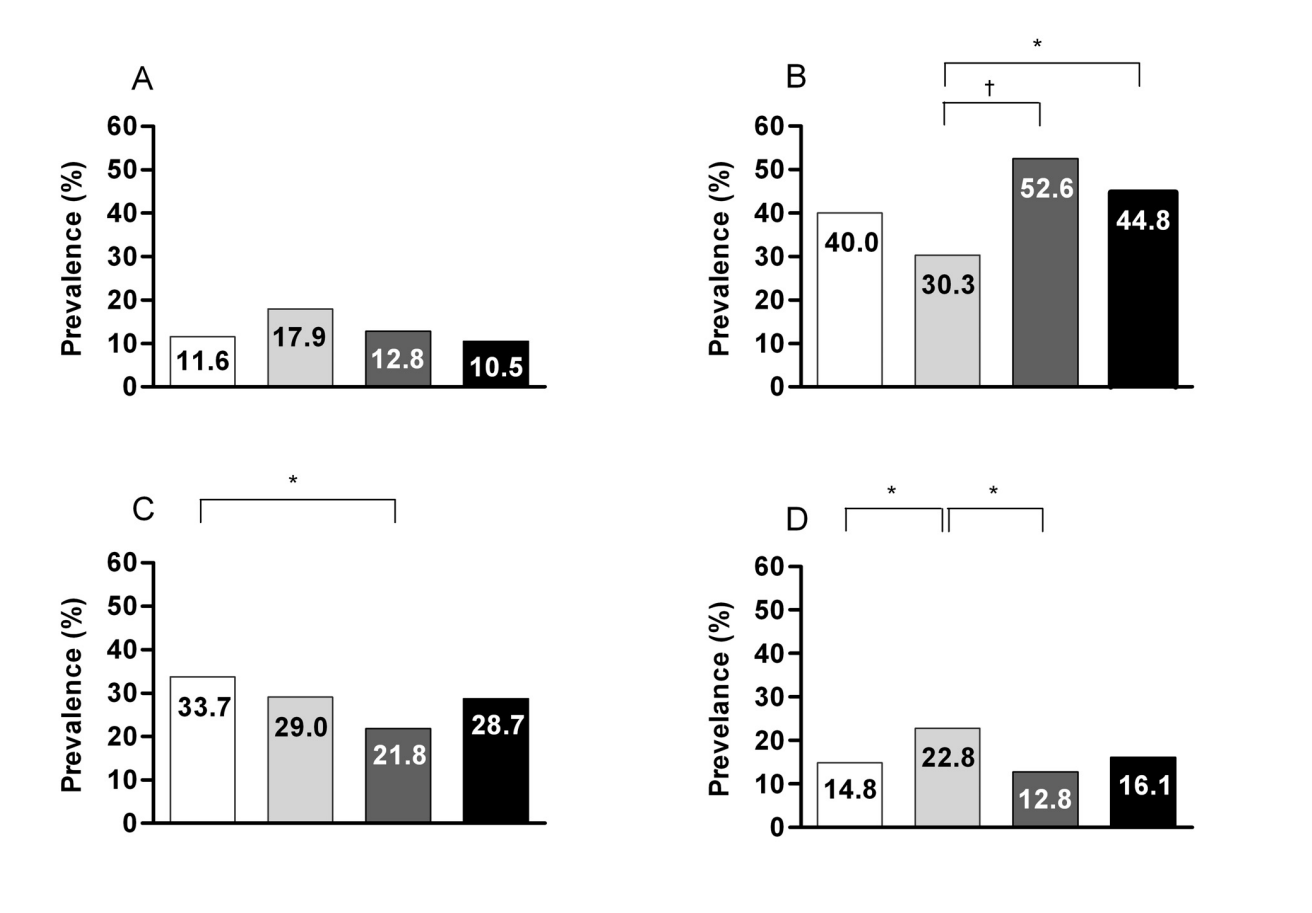
**Prevalences of number of components of the metabolic syndrome; no components (A), one component (B), two components (C), and three or more components (D) according to ethnic group; Dutch native (white bars), Turkish (light grey bars), Moroccan (dark grey bars), and 'other' (black bars)**. * χ^2 ^test, *P *< 0.05. ^† ^χ^2 ^test, *P *< 0.001.

Obesity according to the criterion of MetS (BMI above 97^th ^percentile for age and sex) was present in 83.2% of Dutch native, 69.0% in Turkish, 80.5% in Moroccan and 81.1% in 'other' children. Overall, MetS was present in 16.9%, IGT in 3.2%, low HDL-cholesterol in 31.2%, high triglycerides in 17.4% and hypertension in 22.9% of all children. Only three children were diagnosed with type 2 diabetes. There were no differences in prevalence of MetS or its components when stratified for sex in each of the three ethnic groups, however, MetS and the prevalence of low HDL-cholesterol were more abundant among pubertal children, compared to prepubertal children (20.2% vs. 12.1%, *P *= 0.017 and 35.4% vs. 25.2%, *P *= 0.016, respectively). Figure 1 shows the prevalence of MetS and its different components according to ethnicity. The largest differences were found between the Turkish and Moroccan groups, and these reached statistical significance for MetS, low HDL-cholesterol and hypertension (all *P *< 0.05). High triglycerides tended to be less prevalent in Moroccan children than in Dutch natives (*P *= 0.07). Figure 2 shows the number of components stratified for ethnicity, and reveals no difference in the prevalence of having no MetS-components. When the obesity criterion of MetS was excluded from the analysis, 60.9% of Moroccan children, relative to 48.8% of Dutch native children and 40.0% of Turkish children, were free of MetS-components (*P *= 0.007).

### Impaired fasting glucose, insulin resistance, and high LDL-cholesterol according to ethnicity

Overall, fasting glucose levels of ≥ 5.6 mmol/L were present in 23.1%, and insulin resistance in 48.8% of children. Both were more prevalent in pubertal children, compared to prepubertal children (26.8% vs. 17.8%, *P *= 0.019 and 62.3% vs. 26.1%, *P *< 0.001, respectively). Regarding ethnic-specific differences, highest rates of insulin resistance were found in Turkish children (54.9%), while the prevalence was 49.4% in the 'other' group, 46.8% in the Dutch native group and 37.4% in the Moroccan group, with the difference between the Turkish and Moroccan group being significant (*P *= 0.005) and the difference between the 'other' group and the Moroccan group being borderline significant (*P *= 0.052). In addition, Turkish children had highest HOMA-B levels, in comparison with Moroccan children (Table 1). High LDL-cholesterol (from highest to lowest prevalence) among groups was present in 28.4% of Dutch native children, 19.7% of children with 'other' ethnicities, 14.5% of Turkish children and 10.6% of Moroccan children. The difference between Dutch native children and Moroccan children was significant (*P *= 0.001), as was the difference between Dutch native children and Turkish children (*P *= 0.013) and between the 'other' and the Moroccan group (*P *= 0.044).

### Association of insulin resistance with (components of) the metabolic syndrome and impaired fasting glucose

Table 2 shows the association of insulin resistance (HOMA-IR <3.5 vs. ≥ 3.5) with (components of) MetS and IFG, stratified for the three major ethnic groups, after logistic regression analysis and adjustment for relevant confounders (sex, age and/or Z-BMI). IGT was not included into the analyses due to the very low prevalence, however, insulin resistance was associated with IFG in all ethnic groups. Besides the forementioned association, in Moroccan children, insulin resistance was associated with MetS and its components named in Table 2. In Turkish children, insulin resistance was associated with MetS and low HDL-cholesterol, whereas in the 'other' group, it was associated with MetS and high triglycerides. In the Dutch native group, insulin resistance was only significantly associated with high triglycerides.

**Table 2:** Associations of insulin resistance with (three components of) MetS and IFG

	Dutch Native	Turkish	Moroccan	Other
*n *(%)	95 (18.4)	145 (28.1)	133 (25.8)	145 (27.7)

MetS	2.1 (0.8–6.9)^c^	5.0 (1.7–14.6)*^c^	7.0 (2.1–23.1)*	3.3 (1.2–8.9)^†^

IFG	5.0 (1.5–16.7)*	3.1 (1.3–7.2)*^a^	5.4 (2.3–12.9)*	4.8 (1.6–13.9)*^a, b^

Low HDL-cholesterol	1.4 (0.5–3.5)^c^	4.5 (2.1–9.6)*	3.1 (1.1–8.9)^†b, c^	2.1 (0.9–4.8)^b^

High Triglycerides	3.8 (1.3–10.9)^†^	2.4 (0.9–6.3)^c^	5.2 (1.3–20.4)^†b^	4.1 (1.3–12.9)^†b^

Hypertension	1.2 (0.4–3.5)^a, b, c^	0.7 (0.3–1.6)^b, c^	6.2 (1.8–21.1)*^b^	1.4 (0.5–3.6)^a, b^

Odds ratios (95% confidence intervals) are given for the associations of insulin resistance (binary variable) with the components comprising MetS and IFG (after adjustment for confounders; ^a^sex, ^b^age, ^c^Z-BMI), with logistic regression analysis.

*P < 0.01

^†^*P *< 0.05

HOMA-IR-Homeostasis Model Assessment for Insulin Resistance; MetS-metabolic syndrome; IFG-Impaired Fasting Glucose, IGT-Impaired Glucose Intolerance

## Discussion

This is the first study to compare cardiometabolic risk profiles among different ethnic groups of overweight/obese children living in the Netherlands. In all ethnic groups, a high prevalence of MetS and its components were found, in addition to insulin resistance and high LDL-cholesterol. Highest prevalences of (components of) MetS and insulin resistance were found in Turkish children. In contrast, Moroccan children seem to have a more favourable cardiometabolic risk profile. These findings are in line with the results of a previous Dutch study among adults, in which a higher prevalence of CVD among Turks and a lower prevalence of CVD among Moroccans (living in the Netherlands) were found [7]. In a recent study in children and adolescents by Weiss *et al*, differences in adaptation to insulin resistance among three ethnic groups in the United States were found. It was suggested that obese African American and Hispanic youth had a greater demand on β-cells to maintain glucose levels, while having the same insulin sensitivity, compared to Caucasians [27]. In the present study, we found highest median HOMA-B values in Turkish children, suggesting that β-cell demand was highest within this group. If the former finding would persevere throughout adulthood, it could partially explain the higher prevalence of type 2 diabetes mellitus found among people of Turkish origin living in the Netherlands [9].

We found a markedly lower prevalence of MetS as compared to the study by Weiss *et al*, in children of comparable age, who described a prevalence of 38.7% (moderately obese subjects) to 49.7% (severely obese subjects), applying the same definition [13]. This difference in prevalence of MetS is probably attributable to the different ethnic background and weight status of both cohorts.

Between different ethnic groups among an adult population, a varying prevalence of MetS was found when applying one definition of MetS [28]. In addition, an inconsistent relationship between MetS and CVD was reported, with some populations with low prevalence of MetS carrying a high CVD risk [29]. These discrepancies implicate that different ethnic groups require appropriate reference values in order to establish cardiometabolic abnormalities as well as specific combinations of MetS criteria to predict cardiometabolic risk more reliably, taking into account specific vulnerabilities [29].

There were no statistical significant differences in the percentage pubertal children between the ethnic groups. Moreover, we found similar trends after stratification for pubertal status and ethnic group, however, a formal subanalysis could not be made due to the small size of the subgroups within each ethnic group. We found a similar HOMA-IR in the Turkish and 'other' group, while the 'other' group had a lower percentage of pubertal children in an absolute sense. The former only attributes to the assumption that the degree of insulin resistance is not caused by puberty alone. Although normal puberty is associated with hyperinsulinaemia, it is unclear when this physiological phenomenon becomes pathological i.e. with development of sustained insulin resistance and its related cardiometabolic risk factors [30]. After puberty, the presence of these risk factors may in part resolve, however, one should bare in mind that the presence of MetS during childhood and puberty predicts the presence of MetS and type 2 diabetes in later life [11]. Therefore, the assessment of only insulin resistance parameters in these children may be less reliable to evaluate CVD risk, but rather an overall assessment of cardiometabolic risk factors should be performed in clinical practice. In the logistic regression analyses, pubertal stage was identified as a confounder for the association between insulin resistance and cardiometabolic risk factors. However, since puberty and age are highly correlated, we choose to include age into the analysis, to avoid collinearity of these two variables within the regression analyses. To note, when using puberty instead of age within the models, similar results were found.

In spite of the fact that Turkish children were least likely to fulfil the obesity criterion of MetS according to Dutch-Turkish reference values, they had highest prevalences of cardiometabolic risk factors. This gives rise to the question whether with the use of current reference values, obesity and MetS in this group are underdiagnosed, which would indicate that the prevalence of MetS would even be higher in this group. Some of the differences found may be attributed to environmental factors, such as socio-economic status, lifestyle and diet. However, regarding differences in diet among ethnic groups, its influence may be questioned, due to the fact that both Moroccan and Turkish children have a diet which is more in line with the Dutch guidelines for a healthy diet than the conventional diet as consumed by Dutch native children [31]. The latter does not support the finding that Turkish children have the most unfavourable cardiometabolic risk profile overall, but may account, at least in part, for the relatively low LDL-cholesterol levels in Turkish and Moroccan children. Adaptation of a Western lifestyle after migration is considered by some as major cause of overweight and development of other cardiometabolic risk factors, since the prevalence of these factors is often lower in the country of origin [32]. At least this applies for Turkish adults, but surprisingly, in a cohort of two thousand Moroccan adults living in Morocco, the prevalence of dyslipidaemia and hypertension was respectively three and four fold with respect to Moroccans living in the Netherlands [7,32]. Although confounding factors, such as low socio-economic status and limited access to health care providers in the country of origin may play a role, these findings preclude a reliable assessment of the true contribution of Western lifestyles on the prevalence of cardiometabolic risk factors in Moroccan immigrants.

As shown previously, environmental factors can only partly explain differences found among racial groups, so it is likely that genetic profile plays a key role in expression of obesity related co-morbidities [33], i.e. it is known that the Hindu population in particular carries high risk on diabetes and cardiometabolic risk factors [34]. Genetic influence is also supported by our finding that insulin resistance is differently associated with cardiometabolic risk factors per ethnic group.

In the present study, the group with various 'other' ethnicities revealed significantly higher fasting insulin levels and HOMA-IR as compared to Moroccan children. Additionally, a higher prevalence of cardiometabolic risk factors, such as dyslipidaemia, was found in this group, as compared to Dutch native and Moroccan children. It is likely that the various ethnic groups within this rest-group differ with respect to the prevalence of cardiometabolic risk factors, however, due to the relative small numbers, the exact contribution of each ethnicity to the overall group findings cannot be determined. We suspect that the relatively large number of Hindus within this group caused the high prevalence of cardiometabolic risk factors. The latter emphasizes the importance of ethnic specific risk assessment in these groups.

A limitation of our study is its cross-sectional nature, with data from a single cohort of children in a specific area in the Netherlands. Therefore, our results cannot be extended to the general population. However, we believe that our results may have an important implication given the demographic changes in other parts of the Netherlands. Another limitation is the limited predictive value of our results, since the predictive value of cardiometabolic risk factors in childhood on CVD in adulthood is yet to be established. Also, the contribution of different cardiometabolic risk factors to morbidity and mortality in different racial groups remain largely unknown, and the association of obesity or cardiometabolic risk factors with disease may differ per racial group [35].

## Conclusion

In a Dutch cohort of overweight/obese children, Turkish children showed a higher prevalence of cardiometabolic risk factors relative to their peers of other ethnicities. In contrast, children of Moroccan background seem to have a relatively favourable cardiometabolic risk profile. Our results indicate requirement of an ethnic specific approach in overweight/obese children. Long-term studies are needed to establish whether our findings are compatible with actual risk of future diabetes and CVD in the different ethnic populations.

## List of abbreviations used

MetS: metabolic syndrome; CVD: cardiovascular disease; WC: waist circumference; OR: odds ratio; CI: confidence interval; Z-BMI: Standard deviation score of BMI; Z-WC: Standard deviation score of WC

## Declaration of competing interests

The authors declare that they have no competing interests.

## Authors' contributions

MvV participated in performing the study, data analysis, statistics and writing the manuscript. IAvR contributed to the design of the study, in part performed the study, and reviewed/edited the manuscript. RKS provided methodological and statistical advice, contributed to writing the manuscript and reviewed/edited the manuscript. DPMB contributed to the design of the study, writing the manuscript, and revised/edited the manuscript. JHB contributed in the same ways as DPMB. MD supervised the study and participated in performing the study, the design of the study, data analysis, statistics, and writing the manuscript. All authors read and approved the final manuscript.

## References

[B1] Den Hertog FRJ (2007). Niet-Westerse allochtonen. Volksgezondheid Toekomst Verkenning, Nationale Atlas Volksgezondheid RIVM Rapport.

[B2] Dietz WH (1998). Health consequences of obesity in youth: childhood predictors of adult disease. Pediatrics.

[B3] Must A, Jacques PF, Dallal GE, Bajema CJ, Dietz WH (1992). Long-term morbidity and mortality of overweight adolescents. A follow-up of the Harvard Growth Study of 1922 to 1935. N Engl J Med.

[B4] Sinaiko AR, Donahue RP, Jacobs DR, Prineas RJ (1999). Relation of weight and rate of increase in weight during childhood and adolescence to body size, blood pressure, fasting insulin, and lipids in young adults. The Minneapolis Children's Blood Pressure Study. Circulation.

[B5] Egusa G, Watanabe H, Ohshita K, Fujikawa R, Yamane K, Okubo M, Kohno N (2002). Influence of the extent of westernization of lifestyle on the progression of preclinical atherosclerosis in Japanese subjects. J Atheroscler Thromb.

[B6] Smith SC, Clark LT, Cooper RS, Daniels SR, Kumanyika SK, Ofili E, Quinones MA, Sanchez EJ, Saunders E, Tiukinhoy SD (2005). Discovering the full spectrum of cardiovascular disease: Minority Health Summit 2003: report of the Obesity, Metabolic Syndrome, and Hypertension Writing Group. Circulation.

[B7] Van Leest LATM, van Dis SJ, Verschuren WMM (2002). Hart- en vaatziekten bij allochtonen in Nederland Een cijfermatige verkenning naar leefstijl- en risicofactoren, ziekte en sterfte: RIVM Rapport.

[B8] Schokker DF, Visscher TL, Nooyens AC, van Baak MA, Seidell JC (2007). Prevalence of overweight and obesity in the Netherlands. Obes Rev.

[B9] Garssen J, Bos V, Kunst A, Meulen A van der (2003). Sterftekansen en doodsoorzaken van niet-Westerse allochtonen. CBS Bevolkingstrends.

[B10] Dhuper S, Cohen HW, Daniel J, Gumidyala P, Agarwalla V, St Victor R (2007). Utility of the modified ATP III defined metabolic syndrome and severe obesity as predictors of insulin resistance in overweight children and adolescents: a cross-sectional study. Cardiovasc Diabetol.

[B11] Morrison JA, Friedman LA, Wang P, Glueck CJ (2008). Metabolic syndrome in childhood predicts adult metabolic syndrome and type 2 diabetes mellitus 25 to 30 years later. J Pediatr.

[B12] Viner RM, Segal TY, Lichtarowicz-Krynska E, Hindmarsh P (2005). Prevalence of the insulin resistance syndrome in obesity. Arch Dis Child.

[B13] Weiss R, Dziura J, Burgert TS, Tamborlane WV, Taksali SE, Yeckel CW, Allen K, Lopes M, Savoye M, Morrison J, Sherwin RS, Caprio S (2004). Obesity and the metabolic syndrome in children and adolescents. N Engl J Med.

[B14] Expert panel on Detection, Evaluation and Treatment of High Blood Cholesterol in Adults (Adult Treatment Panel III) (2001). Executive Summary of the third report of the National Cholesterol Education Program (NCEP). JAMA.

[B15] Alberti KG, Zimmet P, Shaw J (2006). Metabolic syndrome – a new world-wide definition. A Consensus Statement from the International Diabetes Federation. Diabet Med.

[B16] Kahn R, Buse J, Ferrannini E, Stern M (2005). The metabolic syndrome: time for a critical appraisal: joint statement from the American Diabetes Association and the European Association for the Study of Diabetes. Diabetes Care.

[B17] Tanner T (1962). Growth at adolescence: with a general consideration of the effects of hereditary and environmental factors upon growth and maturation from birth to maturity.

[B18] Fredriks AM, van Buuren S, Wit JM, Verloove-Vanhorick SP (2000). Body index measurements in 1996–7 compared with 1980. Arch Dis Child.

[B19] Fredriks AM, van Buuren S, Jeurissen SE, Dekker FW, Verloove-Vanhorick SP, Wit JM (2003). Height, weight, body mass index and pubertal development reference values for children of Turkish origin in the Netherlands. Eur J Pediatr.

[B20] Fredriks AM, van Buuren S, Jeurissen SE, Dekker FW, Verloove-Vanhorick SP, Wit JM (2004). Height, weight, body mass index and pubertal development references for children of Moroccan origin in The Netherlands. Acta Paediatr.

[B21] Fredriks AM, van Buuren S, Fekkes M, Verloove-Vanhorick SP, Wit JM (2005). Are age references for waist circumference, hip circumference and waist-hip ratio in Dutch children useful in clinical practice?. Eur J Pediatr.

[B22] NGHS Coordinating Center (1998). NHLBI Growth and Health Study (NGHS) data monitoring report.

[B23] De Man SA, André JL, Bachman H, Grobbee DE, Ibsen KK, Laaser U, Lippert P, Hofman A (1991). Blood pressure in childhood: pooled findings of six European studies. J Hypertension.

[B24] Matthews DR, Hosker JP, Rudenski AS, Naylor BA, Treacher DF, Turner RC (1985). Homeostasis model assessment: insulin resistance and beta-cell function from fasting plasma glucose and insulin concentrations in man. Diabetologia.

[B25] Keskin M, Kurtoglu S, Kendirci M, Atabek ME, Yazici C (2005). Homeostasis model assessment is more reliable than the fasting glucose/insulin ratio and quantitative insulin sensitivity check index for assessing insulin resistance among obese children and adolescents. Pediatrics.

[B26] Friedewald WT, Levy RI, Fredrickson DS (1972). Estimation of the concentration of low-density lipoprotein cholesterol in plasma, without use of the preparative ultracentrifuge. Clin Chem.

[B27] Weiss R, Dziura JD, Burgert TS, Taksali SE, Tamborlane WV, Caprio S (2006). Ethnic differences in beta cell adaptation to insulin resistance in obese children and adolescents. Diabetologia.

[B28] Cameron AJ, Shaw JE, Zimmet PZ (2004). The metabolic syndrome: prevalence in worldwide populations. Endocrinol Metab Clin North Am.

[B29] Banerjee D, Misra A (2007). Does using ethnic specific criteria improve the usefulness of the term metabolic syndrome? Controversies and suggestions. Int J Obes (Lond).

[B30] Bao W, Srinivasan SR, Berenson GS (1996). Persistent elevation of plasma insulin levels is associated with increased cardiovascular risk in children and young adults. Circulation.

[B31] Brussaard JH, van Erp-Baart MA, Brants HA, Hulshof KF, Lowik MR (2001). Nutrition and health among migrants in The Netherlands. Public Health Nutr.

[B32] Tazi MA, Abir-Khalil S, Chaouki N, Cherqaoui S, Lahmouz F, Srairi JE, Mahjour J (2003). Prevalence of the main cardiovascular risk factors in Morocco: results of a National Survey, 2000. J Hypertens.

[B33] Edwards KL, Hutter CM, Yin WJ, Kim H, Monks SA (2008). Genome-wide Linkage Scan for the Metabolic Syndrome: The GENNID Study. Obesity (Silver Spring).

[B34] Bindraban NR, van Valkengoed I, Mairuhu G, Holleman F, Hoekstra JB, Michels BP, Koopmans RP, Stronks K (2008). Prevalence of diabetes mellitus and the performance of a risk score among Hindustani Surinamese, African Surinamese and ethnic Dutch: a cross-sectional population-based study. BMC Public Health.

[B35] Cossrow N, Falkner B (2004). Race/ethnic issues in obesity and obesity-related comorbidities. J Clin Endocrinol Metab.

